# Fetomaternal transfer during Down Syndrome pregnancies induces maternal cognitive decline in an APP‐dependent manner

**DOI:** 10.1002/alz70861_108453

**Published:** 2025-12-23

**Authors:** Eitan Okun, Tamir Hirsh, Ravit Madar, Tomer Illouz

**Affiliations:** ^1^ Bar‐Ilan University, Ramat‐Gan, Ramat‐Gan Israel; ^2^ Bar Ilan University, Ramat Gan, Ramat‐Gan Israel; ^3^ Bar Ilan University, Ramat Gan, Ramat Gan Israel

## Abstract

**Background:**

(1) Establish a mouse model for maternal cogniitive decline following a Down syndrome pregnancy, (2) Determine the underlying mechanism of this phenomenon, (3) Determine the causal factors responsible for this effect, and (4) Assess therapeutic interventions that alleviate maternal cognitive decline.

**Method:**

To model DS‐like early human Amyloid precursor protein (hAPP) overexpression during embryonic stages, wild‐type (WT) female mice were mated with hAPP expressing males (WT/AD females). Following 4 consecutive pregnancies, behavioral‐cognitive assays were done and hAPP levels were traced using genomic, transcriptomic, and proteomic assays, with a control group consisting of WT females mated with WT males (WT/WT females). Adoptive transfer of fetal cells and vaccination strategies were utilized to determine causality and alleviate maternal cognitive decline, respectively.

**Result:**

Elevated levels of human Aβ along with anti‐Aβ IgG were detected in the serum of WT/AD dams. Additionally, we report a short‐ and long‐term memory deficit following 4 pregnancies with either 5xFAD or the APP/PS1 embryos, compared with WT controls. Furthermore, we found elevated levels of hAPP DNA and mRNA transcripts in the brain cortex of WT/AD females, as well as in the liver, whole blood, and mononuclear cells relative to WT/WT mothers, implying that fetal DNA is transcribed, possibly due to intact fetal cell transfer. Also, hAPP and Aβ proteins are found at higher levels in the brain cortex of WT/AD dams. Vaccinating against fetal hAPP epitopes paetially rescused the cognitive deficits of WT/AD dams, while adoptove transfer of fetal cells to pregnant WT dams resulted in impaired cognitive abilities.

**Conclusion:**

Fetomaternal transfer of APP‐related peptides and APP‐expressing cells is responsible for maternal cognitive decline in females carrying APP‐expressing fetuses. this effect can be partially rescued by utilizing vaccination approaches.

